# Insights into ionizing radiation-induced bone marrow hematopoietic stem cell injury

**DOI:** 10.1186/s13287-024-03853-7

**Published:** 2024-07-23

**Authors:** Yimin Zhang, Xinliang Chen, Xinmiao Wang, Jun Chen, Changhong Du, Junping Wang, Weinian Liao

**Affiliations:** 1 State Key Laboratory of Trauma and Chemical Poisoning, Institute of Combined Injury, Chongqing Engineering Research Center for Nanomedicine, College of Preventive Medicine, Army Medical University (Third Military Medical University), Chongqing, 400038 China; 2 Department of Hematology, The General Hospital of Western Theater Command, Chengdu, 610008 Sichuan China; 3 Laboratory of Advanced Biotechnology & State Key Laboratory of Pathogen and Biosecurity, Beijing Institute of Biotechnology, Beijing, 100071 China

**Keywords:** Ionizing radiation, Hematopoietic stem cells, Hematopoietic regeneration, Hematopoietic exhaustion, Hematopoietic aging

## Abstract

With the widespread application of nuclear technology across various fields, ionizing radiation-induced injuries are becoming increasingly common. The bone marrow (BM) hematopoietic tissue is a primary target organ of radiation injury. Recent researches have confirmed that ionizing radiation-induced hematopoietic dysfunction mainly results from BM hematopoietic stem cells (HSCs) injury. Additionally, disrupting and reshaping BM microenvironment is a critical factor impacting both the injury and regeneration of HSCs post radiation. However, the regulatory mechanisms of ionizing radiation injury to BM HSCs and their microenvironment remain poorly understood, and prevention and treatment of radiation injury remain the focus and difficulty in radiation medicine research. In this review, we aim to summarize the effects and mechanisms of ionizing radiation-induced injury to BM HSCs and microenvironment, thereby enhancing our understanding of ionizing radiation-induced hematopoietic injury and providing insights for its prevention and treatment in the future.

## Background

With the widespread application of nuclear energy and nuclear technology in areas such as industrial and agricultural production, military operations, and healthcare, the risk of ionizing radiation-induced injuries is gradually increasing. The bone marrow (BM) serves as the lifelong hematopoietic tissue in the human body and is highly sensitive to ionizing radiation. Thus, BM hematopoietic tissue is a primary target organ for ionizing radiation injury. A certain dose of radiation can cause multi-lineage hematopoietic dysfunction in the BM. Acute radiation syndrome is also mainly characterized by a sharp decrease in whole hematopoietic cells, and results in bleeding, infections, and even death [1].

Hematopoietic stem cells (HSCs) are the primitive cells for all hematopoietic cells, possessing self-renewal and multi-lineage differentiation abilities, which are crucial for maintaining hematopoietic function. However, ionizing radiation-induced injury to BM HSCs leads to severe consequences such as hematopoietic failure, impaired immune function, and hematologic diseases [2]. Therefore, investigating the effects and mechanisms of ionizing radiation-induced injury to BM hematopoiesis, and employing pharmaceutical interventions to alleviate radiation-induced damage to the hematopoietic system, holds significant importance in maintaining health, treating diseases, and saving lives.

With the continuous development in fields such as molecular biology, cell biology, and radiation biology, significant progress has been made in understanding the effects and mechanisms of ionizing radiation-induced injury to BM hematopoiesis. The latest researches have indicated that ionizing radiation directly induces apoptosis, ferroptosis, senescence, and exhaustion of BM HSCs. Additionally, ionizing radiation may also impact hematopoietic tissue injury and regeneration through the disruption of the BM microenvironment. These studies not only contribute to elucidating the impact of ionizing radiation on the BM hematopoietic system, but also provide important theoretical basis and guidance for radiobiology and clinical radiotherapy. In this review, we provide an overview of recent researches on the effects and mechanisms of ionizing radiation-induced injury to BM hematopoiesis, and briefly summarize the impact of ionizing radiation exposure on the injury of BM HSCs and their microenvironment. This may offer new therapeutic strategies for the reconstruction of hematopoietic function following ionizing radiation injury.

## BM HSCs and hematopoietic microenvironment

The BM harbors a rich population of hematopoietic progenitor cells and primitive hematopoietic cells, while HSCs count for less than 0.01% of these cells. However, the hematopoietic function of the BM is maintained by HSCs, which finely balance self-renewal and multi-lineage differentiation to generate all types of hematopoietic cells [3]. Notably, hematopoietic progenitor cells and primitive hematopoietic cells are highly sensitive to radiation, as ionizing radiation-induced DNA damage leading to apoptosis in the majority of these cells. HSCs exhibit a relative insensitivity to radiation, with only a small fraction of cells undergoing apoptosis following low dose radiation exposure. After low-dose radiation injury, survival HSCs generate various hematopoietic cells through proliferation and differentiation, enabling the recovery of hematopoietic function. Nevertheless, lethal doses of radiation can cause severe damage to BM HSCs and impair their self-renewal capacity, resulting in long-term or permanent damage to the hematopoietic system, ultimately leading to hematopoietic function failure and individual death [2]. Therefore, HSC maintenance and regeneration are crucial determinants of the severity and outcome of BM radiation injury. Thoroughly elucidating the regulatory mechanisms governing the fate of BM HSCs post-radiation injury will not only deepen our understanding of the pathogenesis of BM radiation injury but also provide reliable targets for its prevention and treatment.

The homeostasis and survival of BM HSCs are not only regulated by internal factors, but also by numerous external factors in the microenvironment (niche) in which HSCs are located. The hematopoietic microenvironment is primarily composed of progeny hematopoietic cells such as granulocytes, monocytes/macrophages, and T/B lymphocytes, as well as stromal cells such as mesenchymal stem/stromal cells (MSCs), neurons, endothelial cells, and adipocytes [3, 4]. These cells are not only close to HSCs in anatomical position, but they also regulate HSCs homeostasis through intercellular communication, such as secreting cytokines or directly interacting with HSCs [3]. Recent studies have confirmed that radiation also disrupted the HSC niche, leading to extensive apoptosis of niche cells and/or alterations in the secretion profile, which further exacerbated radiation injury to HSCs [5]. Moreover, recent researches have revealed a close association between the microenvironment alterations and the repair of radiation-induced injury to BM HSCs [6–8]. Therefore, ionizing radiation-induced alterations in the BM microenvironment may be a crucial factor influencing the fate determination and homeostasis reshaping of HSCs after radiation injury.

## The effects and mechanisms of ionizing radiation-induced injury to BM HSCs

Acute radiation syndrome, consisting of a series of characteristic clinical manifestations, occurs within a few hours or days after exposure to ionizing radiation. Hematopoietic syndrome entails severe depletion of blood cells resulting from injury or death of BM HSCs, typically occurring at radiation doses of 3–10 Gy in rodents (2–7.5 Gy in humans). Gastrointestinal syndrome occurs after exposure to doses exceeding 5.5 Gy radiation, while neurovascular syndrome occurs following high-dose (exceeding 20 Gy) radiation exposure [2]. This indicates that BM hematopoietic tissue is the most sensitive tissue to ionizing radiation. Moreover, the severity and duration of BM hematopoietic injury are tightly correlated with radiation dose. Exposure to greater than 1 Gy of whole-body radiation typically results in acute and transient BM suppression. Moderate doses (3.5 Gy) of whole-body radiation cause severe damage to BM HSCs, resulting in sustained BM suppression or hematopoietic failure [9].

Moreover, moderate to high doses of whole-body radiation also induces long-term BM hematopoietic injury, characterized by continuously diminished reserve and self-renewal capacity of BM HSCs [10, 11]. Distinguishing from acute hematopoietic injury, the symptoms of radiation-induced long-term BM injury are more insidious. In fact, long-term follow-up observations of radiation-exposed individuals revealed that even after 6 months or longer, despite partial recovery of peripheral blood counts, abnormal hematopoietic function persisted, accompanied by an increased risk of clonal hematopoiesis, immune dysfunction and leukemia, which significantly impacts human health [12]. However, due to the covert nature of its onset, radiation-induced long-term hematopoietic injury has not received adequate attention and lacks comprehensive research.

**Fig. 1:**
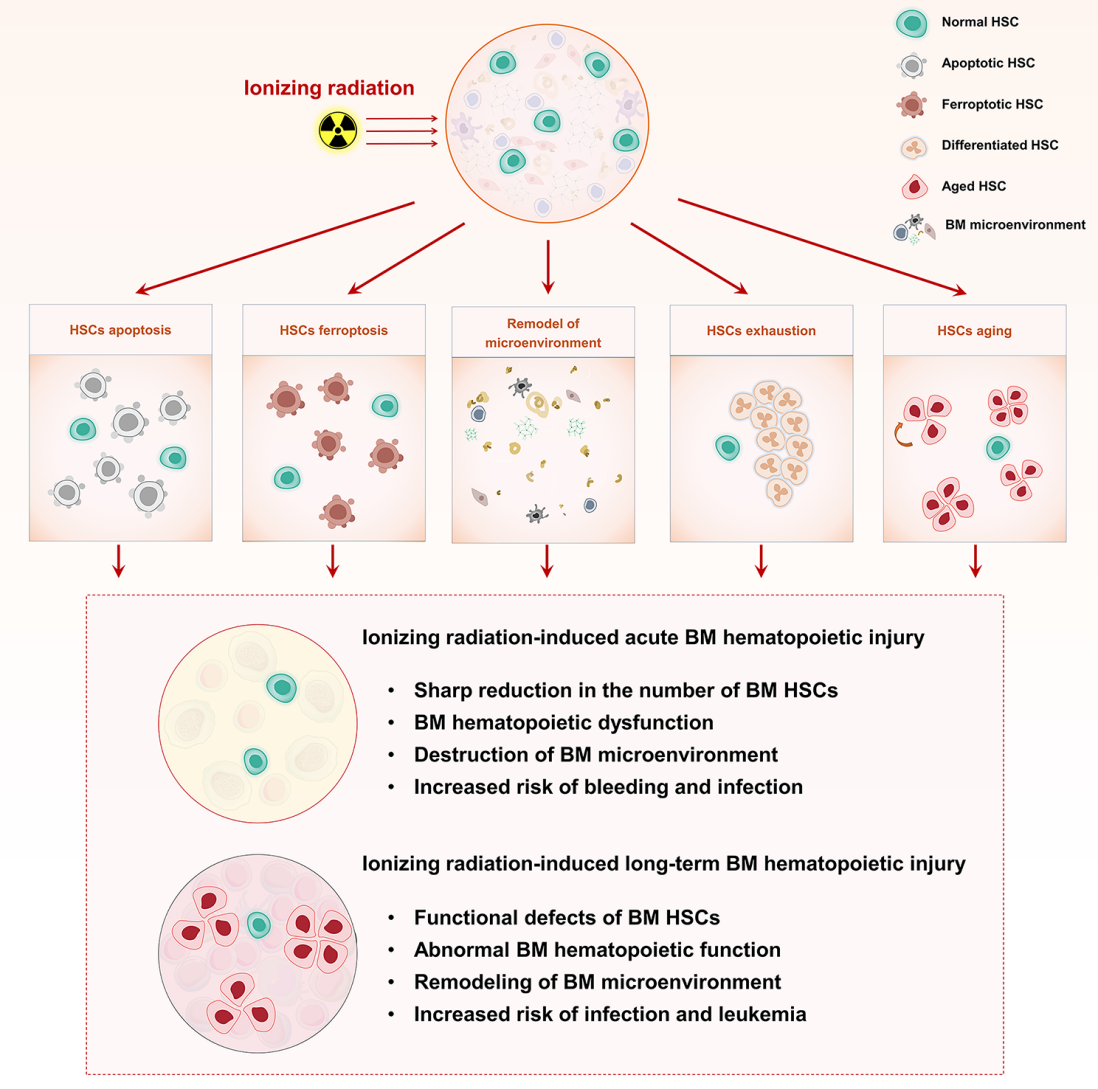
Schematic illustration of effects and mechanisms of ionizing radiation-induced injury to BM HSCs.

Recent researches have proposed several possible explanations for the mechanisms of BM HSCs injury induced by ionizing radiation: (1) Ionizing radiation may induce HSC apoptosis, ferroptosis, or differentiation, leading to a shrink in HSC pool. (2) Ionizing radiation may induce premature aging, resulting in diminished self-renewal and impaired reconstruction capacity of HSCs. (3) Ionizing radiation may induce damage to the HSC niche, thereby affecting HSC regeneration and function (Table 1; Fig. 1). Given that severe damage to BM HSCs resulting in hematopoietic failure is one of the most fatal consequences of moderate- to high-dose radiation injury, understanding the mechanisms of ionizing radiation-induced BM HSC injury will facilitate the development of more targeted clinical prevention and treatment strategies. Therefore, we will discuss these mechanisms in more detail below.

**Table 1:** Effects and mechanisms of ionizing radiation-induced injury to BM HSCs

Radiation conditions	Effect on HSCs	Mechanism	Species	Reference
15 Gy (*ex vivo*)	Apoptosis	Radiation induced persistent DDR foci and p53-dependent apoptosis in HSCs	Human	[16]
8.53 Gy	Apoptosis	Radiation induced the activation of p53 signaling in HSCs, thereby leading to HSC apoptosis	Mouse	[18]
0–6 Gy (*ex vivo*)	Apoptosis	Anti-apoptotic protein Bcl-2 was essential in protecting HSCs from the radiation-induced apoptosis	Mouse	[19]
5 Gy	Apoptosis	Radiation induced mitochondrial ROS production and mitochondrial damage, which promoted HSCs apoptosis	Mouse	[20]
4, 7.5, 9.5 Gy	Apoptosis	Radiation induced significant ROS production, DNA damage, and thereby promoting apoptosis in HSCs	Mouse	[21]
5, 7.5 Gy	Ferroptosis	Radiation-induced redox imbalance including increased Fe2^+^ pool, decreased GSH/GSSG ratio, and ACSL4 upregulation may lead to ferroptosis of HSCs	Mouse	[26]
5 Gy	Ferroptosis	Radiation induced alterations in mitochondrial oxidative metabolism and autophagy may increase the ferroptosis vulnerability of HSCs	Mouse	[28]
0.1, 0.25, 1 Gy	Activation and exhaustion	Radiation induced increased ROS production may cause DNA damage and stimulated activation of quiescent HSCs	Mouse	[32]
20 mGy	Activation and exhaustion	Low-dose radiation induced a rapid and transient increase of ROS that promotes activation of HSC via p38MAPK pathway	Human	[34]
6 Gy	Activation and exhaustion	SRC-3 rescued radiation-induced HSC activation by inhibiting abnormal mitochondrial activation and excessive production of ROS	Mouse	[35]
7.5 Gy	Activation and exhaustion	SREBF1c restrained radiation-induced HSC activation by inhibiting the excessive activation of mTOR signaling and mitochondrial metabolism	Mouse	[36]
7 Gy	Premature aging	Radiation induced HSC senescence through ROS activated GCN2/eIF2α/ATF4 signaling pathway	Mouse	[10]
5 Gy	Premature aging	Radiation induced HSC aging and reprogrammed microenvironment conferred a prosurvival niche for aged HSCs	Mouse	[11]
6.5 Gy	Premature aging	Radiation induced HSC senescence through NADPH oxidase 4 -mediated ROS prodaction	Mouse	[33]
6 Gy	Premature aging	Radiation-induced DNA damage activated the p38 MAPK cascade may induce HSC senescence	Mouse	[38]

### Ionizing radiation-induced apoptosis of BM HSCs

Apoptosis refers to the active and physiological process of cell death under certain physiological or pathological conditions, controlled by internal genetic mechanisms, according to its own program [13]. Apoptosis, coordinating with proliferation and differentiation, contributes to the maintenance of BM hematopoietic homeostasis by precisely regulating the pool size of HSCs. However, dysregulation of HSC apoptosis may lead to various hematological diseases such as aplastic anemia, myelodysplastic syndrome and leukemia [14]. Increasing evidence suggests that radiation damages HSCs by inducing apoptosis. Firstly, radiation has been confirmed as a potent inducer of various cell apoptosis, as radiation directly causes damage to chromosomal DNA. Radiation-induced DNA damage triggered the activation of the intracellular DNA damage response mechanism, including the phosphorylation of proteins such as Ataxia telangiectasia mutated (ATM) and Ataxia telangiectasia and Rad3 related (ATR), as well as an increase in the expression of key proteins regulating the cell cycle and apoptosis, such as p53 and p21, ultimately leading to HSC apoptosis [15, 16]. Consequently, the loss of the critical apoptosis-regulating protein p53 significantly reduced the sensitivity of mouse HSCs to ionizing radiation. Treatment with p53 inhibitors could protect mice from radiation-induced lethal damage by inhibiting p53-dependent apoptotic signaling [17]. Prostaglandin E2 also blocked the activation of p53 signaling in HSCs, thereby reducing HSC apoptosis and achieving therapeutic efficacy against lethal radiation [18]. Similarly, high expression of the anti-apoptotic protein B-cell lymphoma-2 (Bcl-2) in the hematopoietic system protected mice from the adverse effects of radiation, such as hematopoietic failure and death. HSCs isolated from Bcl-2 transgenic mice exhibited increased resistance to radiation damage in vitro. Conversely, Bcl-2 deficiency increased the sensitivity of BM HSCs to radiation injuries [19].

Furthermore, the generation of free radicals and oxidative stress induced by ionizing radiation is also a significant mechanism leading to HSC apoptosis [15]. By inhibiting mitochondrial oxidative damage to reduce the generation of reactive oxygen species (ROS), or by enhancing the activity of glutathione peroxidase to facilitate the clearance of ROS, both approaches alleviated radiation-induced HSC apoptosis and hematopoietic dysfunction [20, 21]. Otherwise, radiation-induced bystander effect also increased oxidative stress in HSCs, leading to DNA damage response and p53-dependent apoptosis [22]. Therefore, apoptosis of BM cells, including HSCs, may be one of the primary causes of acute radiation syndrome in the BM.

### Ionizing radiation-induced ferroptosis of BM HSCs

Ferroptosis is a specific form of cell death caused by iron-dependent lipid peroxidation. Although ionizing radiation has been demonstrated to induce ferroptosis in cells [23], and iron chelators have proven therapeutic effects on radiation-induced intestinal diseases [24], relatively little attention has been given to the phenomenon of radiation-induced ferroptosis in HSC. Recent studies have revealed that the loss of glutathione peroxidase 4 (GPX4), a crucial regulatory protein in ferroptosis signaling, leads to an increase in lipid peroxidation and cell death in HSCs following radiation damage [25]. Our research has shown that, in the early stages after ionizing radiation (approximately 24 h), BM HSCs exhibit typical signs of ferroptosis, including increased cell death, elevated levels of lipid peroxidation, and an expansion of the Fe^2+^ pool, along with a disruption in the redox homeostasis [26]. Interestingly, radiation-induced ferroptosis in HSCs persisted from 24 h after exposure to radiation up to 72 h or even longer [26], suggesting that apart from the direct injury caused by radiation, there may be other factors inducing HSC ferroptosis in the BM microenvironment during the post-radiation period.

Indeed, ferroptosis is regulated by multiple signaling pathways and various cellular metabolic pathways, including iron metabolism, energy metabolism, as well as amino acid, lipid, and glucose metabolism [27]. Considering the unique lipid metabolism characteristics of HSCs, we found that cholesterol supplement significantly reduced the sensitivity of HSCs to ionizing radiation-induced ferroptosis [26]. Mechanistically, cholesterol supplement enhanced the ferroptosis resistance of HSC by activating the SLC38A9-mTOR signaling axis which upregulated SLC7A11/GPX4 expression and inhibited iron autophagy [26]. Furthermore, mTOR downstream of IGF1 signaling also inhibits ferritinophagy to restrict radiation-induced HSC ferroptosis [28]. Another research has reported that the loss of the histone deubiquitinase Myb-Like SWIRM and MPN domains 1 (MYSM1) increased the sensitivity of HSCs to ferroptosis by reducing protein synthesis rates in HSCs [29]. This suggests that radiation may preferably induce ferroptosis a selectively disadvantaged HSC population with lower protein synthesis, further confirming significant diversity in ferroptosis sensitivity among HSC subpopulations. Therefore, radiation-induced ferroptosis is an important mechanism of BM HSC injury and a key factor determining the survival advantage of HSC subpopulations and lineage reconstruction during hematopoietic regeneration following radiation injury.

### Ionizing radiation-induced abnormal activation and exhaustion of BM HSCs

Under normal circumstances, the proportion, quantity, proliferation, and differentiation fate of HSCs are maintained in a relatively stable state, with cellular quiescence being a prerequisite for maintaining this equilibrium. The maintenance of HSCs in a quiescent state is closely linked to their unique energy metabolism pattern [30]. However, unlike other cells, HSCs reside in a relatively hypoxic microenvironment within the BM, causing their energy metabolism to primarily rely on anaerobic glycolysis, with aerobic metabolism being relatively inhibited. The energy metabolism pattern thus sustains the quiescent state of HSCs [30]. However, exposure to ionizing radiation prompts HSCs to exit their quiescent state and mobilize from the BM or undergo proliferation and differentiation through endogenous factors and microenvironmental regulation, thus accelerating the regeneration and repair of BM hematopoietic tissue [31]. However, sustained exposure to radiation-induced oxidative stress and a shift in energy metabolism pattern may also disrupt the homeostasis of murine HSCs and lead to their continues proliferation, differentiation, and ultimately exhaustion [32, 33]. Similarly, radiation reduces the reconstitution potential of human HSCs through a rapid and transient increase of ROS/p38MAPK pathway [34]. Therefore, besides inducing apoptosis and ferroptosis in HSCs, exposure to relatively high doses of radiation also disrupts the quiescent state of HSCs, leading to their excessive mobilization or differentiation, ultimately resulting in HSC exhaustion.

We have found that mitochondrion was a crucial target for the imbalance and exhaustion of BM HSCs induced by ionizing radiation. Activation of the steroid receptor coactivator-3 (SRC-3) [35]involved in inhibiting abnormal mitochondrial activation by regulating the acetyltransferase general control non-depressible 5 (GCN5) activity, and the sterol regulatory element binding factor-1c (Srebf1c) [36] also suppressed abnormal mitochondrial activation by regulating the Tuberous sclerosis complex 1 (TSC1)/ mTOR complex 1 (mTORC1) signaling pathway activity, both rescuing radiation-induced HSC exhaustion. Additionally, in our investigations focused on modulating cell energy metabolism in the context of radiation injury, we found that antioxidants such as caffeic acid [20] and grape seed proanthocyanidin extract [21], by activating 5-lipoxygenase (5-LOX) and forkhead Box O1 (FoxO1), respectively, alleviated mitochondrial metabolic disturbances in BM HSCs and mitigated HSC exhaustion after radiation injury.

It is noteworthy that different mechanisms exist for the disturbing and reshaping of HSC homeostasis at different stages after radiation injury. On one hand, radiation-induced DNA damage activated the intracellular DNA damage response mechanism, potentially leading to cell cycle arrest, exhaustion of repair mechanisms, and impairment of self-renewal function in HSCs [18]. On the other hand, during the BM radiation injury repair process, continuous activation of the cell cycle in BM HSCs may also contribute to HSC exhaustion [37]. Therefore, precise regulation of HSC homeostasis after radiation injury is essential to effectively promote sustained HSC output, minimize HSC exhaustion, and concurrently reduce the incidence of leukemia.

### Ionizing radiation-induced premature aging of BM HSCs

Ionizing radiation not only causes acute injury to BM hematopoiesis, but also causes long-term injury to BM HSCs. After whole-body exposure to relatively high-dose radiation, BM HSCs of mice gradually exhibit signs like physiological aging. Radiation-induced premature aging of BM HSCs is characterized by elevated levels of aging markers (such as SA-β-gal, p16, and Arf), impaired self-renewal capacity, diminished long-term reconstruction ability, and increased myeloid-biased differentiation, accompanied by a decline in overall immune and anti-infection capabilities [2, 10, 11]. Of note, the excessive myeloid-biased differentiation of HSCs may contribute to abnormal HSC proliferation, potentially leading to the development of myeloid leukemia [2]. However, the effects and mechanisms by which radiation induces premature aging of BM HSCs remain unclear.

Early studies suggested that ionizing radiation might induce mitochondrial damage, leading to increased ROS generation in HSCs. ROS, in turn, could induce HSC premature aging by stimulating the p38 pathway or activating the ATM/Checkpoint Kinase 2 (CHK2)/p53/p21 pathway in response to radiation-induced DNA damage [2, 33, 38]. As a complement, our recent research indicated that long term post radiation, HSCs exhibited obvious suppression of apoptosis signaling similar to physiological aging. Triggering apoptosis signals significantly corrected the aging phenotype and functionality of BM HSCs after long term post radiation [11]. Therefore, the reduced apoptosis sensitivity is a crucial factor contributing to the enhanced clonal advantage and decreased hematopoietic reconstruction ability in prematurely aged HSCs after exposure to radiation.

## The effects and mechanisms of ionizing radiation-induced injury to the BM microenvironment

Ionizing radiation, apart from directly damaging BM HSCs, also leads to BM microenvironment injuries. Radiation-induced disruption of the BM microenvironment can exacerbate the damage to BM HSCs and hinder the repair of BM hematopoietic tissue in various ways. In the early stages following radiation injury, there is a rapid adjustment and repair of cell proportions, functional activity, and interactions within the BM, leading to the disruptions of the microenvironment. The reshaped microenvironment provides survival and proliferation signals for HSCs, aiding in the restoration of BM hematopoietic regenerative capacity. However, in the long term after radiation injury, the reshaped BM microenvironment also leads to long-term hematopoietic injury. Therefore, in-depth research into the impacts of radiation on the BM microenvironment and underling mechanisms is crucial for developing more effective treatment strategies and improving the efficiency of BM hematopoietic regeneration after radiation exposure (Table 2).

**Table 2:** Effects and mechanisms of BM HSCs impacted by ionizing radiation-induced microenvironment (niche) alterations

Niche factors (niche cells)	Effect	Mechanism	Species	Ref
CXCL12, IL-7 and KITL (CD73^+^NGFR^hi^ stromal cells)	Promoting HSC regeneration	CD73^+^NGFR^hi^ stromal cells expressed high levels of cytokines which promoted early hematopoietic regeneration post radiation	Mouse	[41]
IGF1 (megakaryocytes)	Promoting HSC regeneration	Megakaryocytes became a predominant component of HSC niche and were characterized by predominant and reversible IGF1 hypersecretion, which is necessary for optimal HSC regeneration	Mouse	[28]
VEGF (LepR^+^ stromal cells)	Promoting HSC regeneration	VEGF derived from LepR^+^ stromal cells improves hematopoietic recovery after radiation by accelerating endothelial and LepR^+^ cell regeneration	Mouse	[6]
SCF (adipocytes)	Promoting HSC regeneration	Adipocytes expressed a high level of SCF which promoted the HSC regeneration post radiation	Mouse	[8]
PDGF-BB (megakaryocytes)	Promoting HSC regeneration	Megakaryocytes stimulated the proliferation of osteoblasts via PDGF-BB, thus promoting hematopoietic regeneration	Mouse	[42]
VEGF (macrophages)	Promoting HSC regeneration	Macrophages sensed and responded to structural changes in the BM microenvironment post radiation, secreting VEGF to promote microvascular neogenesis, thereby facilitating HSC regeneration	Mouse	[45]
G-CSF (nociceptor neurons)	Promoting HSC regeneration	Nociceptor neurons drove G-CSF secretion induced HSC mobilization via the secretion of calcitonin gene-related peptide	Mouse	[46]
Extracellular adenosine (Tregs)	Promoting HSC regeneration	Paracrine secretion of adenosine derived from Tregs promoted hematopoietic regeneration post radiation	Mouse	[52]
Extracellular ATP	Exacerbating long-term HSC injury	Radiation induced elevated levels of ATP in the BM microenvironment. ATP triggered P2X7 receptor signaling to mobilize and differentiate HSCs which contribute to long-term deleterious on hematopoiesis	Mouse	[55]
cAMP (Tregs)	Exacerbating long-term HSC injury	Tregs facilitated the transfer of cAMP through gap junctions to aged HSCs, activating the PKA-CREB signaling pathway and reducing the apoptosis sensitivity of HSCs, thereby promoting HSC aging in the long term post radiation	Mouse	[11]
IL-1, IL-18	Exacerbating long-term HSC injury	Secretion of inflammatory cytokines such as IL-1 and IL-18 promoted the expansion of BM macrophages and CD8^+^ T cells, thus leading to the destruction of the BM microenvironment and depletion of HSCs in the long term after radiation	Mouse	[56]

### Ionizing radiation causes damage to the BM microenvironment

Initially, ionizing radiation demonstrates varied detrimental effects on distinct cellular populations within the BM microenvironment, resulting in a significant reduction in the abundance of most BM cells. For instance, BM stromal cells were highly sensitive to radiation, inducing dose- and time-dependent damage to these cells. The repair of BM stromal cells was crucial for initiating hematopoietic reconstruction [39]. BM endothelial cells were also highly sensitive to radiation. Radiation-induced the secretion of semaphorin 3 A (SEMA3A) by BM endothelial cells, which, through its receptor neuropilin 1 (NRP1), triggered endothelial cell apoptosis, thereby disrupting the integrity of the BM microvasculature [7]. Additionally, there was a significant increase in osteoclast proportions within the BM post-radiation, accompanied by a marked decrease in bone volume and noticeable disruption of the extracellular matrix structure within the bone [40]. Consequently, radiation exposure disrupts the pre-existing structure and homeostasis of the BM microenvironment, leading to complex impacts on the survival and regeneration of HSCs.

### Ionizing radiation-induced microenvironment disruptions promote early hematopoietic regeneration

Remodeled BM microenvironment caused by ionizing radiation also provides an important foundation for early BM hematopoietic tissue regeneration and repair after radiation injury. Research has found that after radiation injury, a small subset of BM stromal cells, such as CD73^+^NGFR^hi^ chondrocytes, survived. These cells promoted BM hematopoietic regeneration after radiation injury by secreting cytokines like CXCL12, IL-7, and KITL [41]. Megakaryocytes have been found to be resistant to even lethal radiation, and megakaryocytes become a predominant component of HSC niche and elicit an adaptive response that is characterized by predominant and reversible IGF1 hypersecretion, which is necessary for optimal HSC regeneration [28]. Endothelial cells, adipocytes, and other cell types enhanced the hematopoietic regeneration capacity of surviving HSCs in the BM post-radiation injury by secreting hematopoietic growth factors such as vascular endothelial growth factor (VEGF) and stem cell factor (SCF) [6, 8]. Similarly, surviving BM megakaryocytes during the early stage after radiation exposure stimulated the proliferation of BM stromal cells, such as osteoblasts via platelet-derived growth factor-BB (PDGF-BB) [42]. However, the early recovery of osteoblasts came at the cost of “sinusoidal dilation and congestion” [43]. Furthermore, radiation disturbed the proliferation and differentiation capacity of murine mesenchymal stem cells, leading to a decreased osteogenic differentiation potential and an increased adipogenic differentiation potential [44]. The increase in adipocyte numbers post-radiation served as a significant source of stem cell factors, exerting a positive regulatory effect on HSC regeneration [8]. After exposure to radiation, BM-resident macrophages undergo significant activation. Activated macrophages sensed and responded to structural changes in the BM microenvironment post-radiation injury, secreting VEGF to promote microvascular neogenesis, thereby facilitating hematopoietic regeneration [45]. Moreover, the adrenergic nervous system from the sympathetic nervous system modulated the mobilization and hematopoietic recovery of HSCs by regulating the secretion of granulocyte colony-stimulating factor (G-CSF), promoting the maintenance of HSC regenerative capacity and multi-lineage differentiation potential [46, 47]. B cells, serving as essential sources of the parasympathetic nervous system neurotransmitter acetylcholine, influenced HSC regeneration by regulating the expression of acetylcholine receptors in endothelial cells in the BM microenvironment and the secretion of CXCL12 by mesenchymal stromal cells [48].

Furthermore, BM HSCs reside in an immunosuppressive microenvironment to evade immune-mediated damage. Exposure to radiation similarly leads to reshaping of the BM immune microenvironment, where immune cells within the microenvironment promote the regeneration of BM hematopoietic tissue through various mechanisms. Regulatory T cells (Tregs), as crucial immunoregulatory components of the body, play pivotal roles in the formation of the immunosuppressive microenvironment for stem cells. Positioning anatomically close to stem cells, Tregs maintain the immune exemption status of stem cells through direct contact or secretion of immunosuppressive factors [49, 50]. Recent studies have also found that Tregs were very close to HSCs in normal BM tissue, where Tregs maintained the homeostasis and immune privilege status of HSCs through paracrine secretion of adenosine, IL-10, and other factors [51, 52]. Moreover, Tregs are relatively insensitive to radiation, leading to their significant survival post-radiation injury, which consequently increases their proportion in the BM [11]. Following exposure to lethal doses (9 Gy) of radiation, depletion of BM Tregs further reduced HSC survival, while adoptive Treg transfer promoted HSC survival and effectively alleviated radiation-induced BM suppression [53]. These observations suggest that the increased survival of Tregs in the BM HSC microenvironment following radiation injury contributes to the maintenance of HSC survival, although the specific processes, links, and mechanisms involved require further elucidation.

### Ionizing radiation-induced microenvironment alterations exacerbate long-term hematopoietic injury

Although ionizing radiation-induced reshaping of the BM microenvironment may promote BM tissue repair in the early stages following radiation injury, such microenvironmental alterations may also mediate long-term injury to BM hematopoiesis. For instance, while increased adipogenic differentiation capacity of murine BM mesenchymal stem cells enhanced early hematopoietic regeneration post-radiation injury, alterations in their differentiation fate also accelerated accumulation of adipose tissue in the long term after radiation, thereby compressing the spatial distribution of BM hematopoietic cells [54]. Exposure to radiation also induced elevated levels of danger signaling molecules such as ATP in the BM microenvironment. ATP triggered P2X7 receptor signaling to mobilize and differentiate HSCs, thereby promoting early BM hematopoietic regeneration after injury. However, sustained activation of P2X7 signaling also induced excessive mobilization and differentiation of HSCs, accelerating their depletion [55].

Similarly, ionizing radiation-induced reshaping of the BM immune homeostasis also leads to long-term injury to BM hematopoiesis. In the long term after radiation exposure, DNA mutations persistently accumulated in BM HSCs, subsequently inducing an upregulation of surface expression of major histocompatibility complex class II (MHCII) molecules [11]. The upregulation of MHCII expression increased the chances of recognition between aged HSCs and BM Tregs, leading to the clonal expansion of Tregs and their accumulation in the hematopoietic microenvironment. Furthermore, Tregs further facilitated the transfer of cyclic adenosine monophosphate (cAMP) through gap junctions to aged HSCs, activating the protein kinase A (PKA)/cAMP-response element-binding protein (CREB) signaling pathway and reducing the apoptosis sensitivity of HSCs. This ultimately results in the enhanced clonal dominance of Tregs. Conversely, interventions such as targeted clearance of Tregs and the utilization of gap junction inhibitor GAP27 significantly eliminate aged HSCs, reducing the accumulation of genetic mutations in HSCs and ameliorating long-term BM hematopoietic injury induced by radiation [11]. These results also confirm the crucial role of the interaction between BM Tregs and HSCs in the context of radiation-induced long-term hematopoietic injury. Additionally, local radiation-induced reshaping of the BM immune microenvironment may also exert long-term detrimental effects on distal BM hematopoietic tissues. Research indicates that local radiation stimulates the secretion of inflammatory cytokines such as IL-1 and IL-18, promoting the expansion of BM macrophages and CD8^+^ T cells, thus leading to the destruction of the BM microenvironment and depletion of HSCs in the long term after radiation [56].

## Translational implications

Research on the effects and mechanisms of ionizing radiation-induced damage to BM HSCs has opened new directions for the treatment of hematopoietic radiation injury and hematopoietic system disorders. Firstly, diminishing apoptosis and ferroptosis signaling pathways significantly reduces the depletion of BM HSCs early after radiation, thereby promoting recovery and reconstruction of hematopoietic tissues [18, 20, 21, 26, 28]. Moreover, targeting the susceptibility gene, inflammation or energy metabolism signaling pathways can effectively mitigate radiation-induced activation and depletion of HSCs [20, 21, 35, 36]. Although suppressing the death and depletion of HSCs early post-radiation exposure indeed accelerates hematopoietic regeneration, further research is needed to clarify whether surviving HSCs harbor radiation-induced genetic mutations that could accumulate in their daughter cells [57]. It is noteworthy that besides the treatment of radiation-induced acute injury to BM HSCs, managing the radiation-induced long-term effects is equally crucial. Therapeutic measures such as regulating redox balance or depleting aged HSCs have been shown to effectively reverse the premature aging phenotypes in both HSCs and hematopoietic cells [10, 11, 33]. However, whether these measures have therapeutic effects on the dysfunctions of hematopoietic and immune systems in the long term post radiation remains to be determined. Furthermore, apart from directly damaging HSCs, radiation also indirectly impacts hematopoietic reconstruction by disrupting the BM microenvironment. Therefore, protecting the survival and function of niche cells, such as stromal cells, adipocytes and megakaryocytes—also provides potential strategies for treating radiation-induced injury to BM HSCs [6, 8, 28, 41, 42].

## Conclusions

Nuclear accidents or nuclear explosions result in high mortality and disability rates due to ionizing radiation injury, posing significant challenges for prevention and treatment. The BM serves as the lifelong hematopoietic tissue in the body and is a primary target organ for ionizing radiation injury. Exposure to a certain dose of ionizing radiation caused hematopoietic dysfunction in the BM. Previous researches have confirmed that radiation damage to BM HSCs is the pathological basis for radiation-induced hematopoietic dysfunction. Ionizing radiation directly induces damage to HSCs through mechanisms such as apoptosis, ferroptosis, exhaustion, and premature aging. Additionally, it indirectly affects the HSC maintenance and regeneration by impacting the BM microenvironment. Ultimately, ionizing radiation may lead to BM hematopoietic failure and organismal death by weakening the reconstruction function of HSC hematopoiesis or depleting the quantity of HSCs. Bleeding and infection resulting from BM HSCs exhaustion are primary causes of mortality in individuals exposed to high doses of ionizing radiation. Consequently, safeguarding BM HSCs from radiation-induced injury has become a primary goal in clinical management of radiation injuries. However, due to the high heterogeneity of HSC populations and the complexity of the BM microenvironment, significant differences exist in the cellular states, radiation sensitivity, gene expression regulation, and intercellular communication of various HSC subpopulations and microenvironment cell populations. This greatly increases the difficulty and complexity of research in this field. However, with the continuous advancement and iteration of technologies such as single-cell sequencing, immunology, proteomics, metabolomics, our understanding of the effects and mechanisms of ionizing radiation-induced BM hematopoietic injury is accelerating. In the future, targeting the regulation of HSC apoptosis, ferroptosis, mitochondrial energy metabolism, and the BM microenvironment holds promise as new strategies for preventing and treating radiation injury. This will provide a more scientifically guided approach for clinical prevention and treatment of BM hematopoietic radiation injury.

## Data Availability

Not applicable.

## Abbreviations

ATM: Ataxia telangiectasia-mutated
ATR: Ataxia telangiectasia and Rad3 related
Bcl-2: B-cell lymphoma-2
BM: Bone marrow
CHK2: Checkpoint Kinase 2
cAMP: Cyclic adenosine monophosphate
CREB: cAMP-response element-binding protein
FoxO1: Forkhead Box O1
G-CSF: Granulocyte colony-stimulating factor
GCN5: general control non-depressible 5
GPX4: Glutathione peroxidase 4
HSC: Hematopoietic stem cell
MHCII: Major histocompatibility complex class II
MSCs: mesenchymal stem/stromal cells
mTORC1: mTOR complex 1
MYSM1: Myb-Like SWIRM and MPN domains 1
NRP1: Neuropilin 1
ROS: Reactive oxygen species
SCF: stem cell factor
SEMA3A: Semaphorin 3 A
SRC-3: Steroid receptor coactivator-3
Srebf1c: Sterol regulatory element binding factor-1c
Tregs: Regulatory T cells
TSC1: tuberous sclerosis complex 1
PKA: protein kinase A
PDGF-BB: Human Platelet-Derived Growth Factor-BB
VEGF: Vascular endothelial growth factor
5-LOX: 5-Lipoxygenase
